# IntReALL HR and the benefits of adaptive design

**DOI:** 10.1186/1745-6215-16-S2-P216

**Published:** 2015-11-16

**Authors:** Jane Holmes, Sharon Love, Vaskar Saha, Arend von Stackelberg

**Affiliations:** 1 Oxford University, Oxford, Oxfordshire, UK; 2 Manchester Academic Health Sciences Centre, Manchester, UK; 3 University Medicine Berlin, Berlin, UK

## 

Survival of children with acute lymphoblastic leukemia (ALL) has improved considerably over the past few decades, but relapsed ALL remains difficult to treat, particularly in those whose relapse puts them in a high risk (HR) group. Little progress has been made during the last two decades in the HR group despite several new drugs being investigated.

The International BFM Study Group consists of experts from 20 European countries who have combined to ensure enough patients to carry out clinical trials and advance the treatment of HR relapsed ALL paediatric patients. Even though the previous 2 new treatments have not been beneficial, these clinicians are keen to give patients new treatments that look promising and are reticent to recruit them into a trial where there is only a 50% chance of being randomised to the new treatment. Hence the design chosen for IntReALL-HR is a covariate adaptive response adaptive (CARA) randomised controlled trial of bortezomib plus standard treatment versus standard treatment alone in children with relapsed ALL in the HR group. Patients are randomised with higher probability to the treatment that is currently “best”, thereby gaining the benefits of randomisation, while satisfying the clinicians in assigning more patients to the better treatment and being a benefit to the patients who have a high chance of obtaining the best treatment at the point of their diagnosis with relapse. All countries have agreed to take part in the trial.

